# Microenvironmental niche divergence shapes BRCA1-dysregulated ovarian cancer morphological plasticity

**DOI:** 10.1038/s41467-018-06130-3

**Published:** 2018-09-25

**Authors:** Andreas Heindl, Adnan Mujahid Khan, Daniel Nava Rodrigues, Katherine Eason, Anguraj Sadanandam, Cecilia Orbegoso, Marco Punta, Andrea Sottoriva, Stefano Lise, Susana Banerjee, Yinyin Yuan

**Affiliations:** 1 Centre for Evolution and Cancer, The Institute of Cancer Research, London, SM2 5NG UK; 2 Division of Molecular Pathology, The Institute of Cancer Research, London, SM2 5NG UK; 3 Division of Cancer Therapeutics, The Institute of Cancer Research, London, SM2 5NG UK; 4 Centre for Molecular Pathology, Royal Marsden Hospital, London, SM2 5NG UK; 5 Gynaecology Unit, The Royal Marsden NHS Foundation Trust, London, SW3 6JJ UK; 6 Division of Clinical Studies, the Institute of Cancer Research, London, UK SM2 5NG

## Abstract

How tumor microenvironmental forces shape plasticity of cancer cell morphology is poorly understood. Here, we conduct automated histology image and spatial statistical analyses in 514 high grade serous ovarian samples to define cancer morphological diversification within the spatial context of the microenvironment. Tumor spatial zones, where cancer cell nuclei diversify in shape, are mapped in each tumor. Integration of this spatially explicit analysis with omics and clinical data reveals a relationship between morphological diversification and the dysregulation of DNA repair, loss of nuclear integrity, and increased disease mortality. Within the Immunoreactive subtype, spatial analysis further reveals significantly lower lymphocytic infiltration within diversified zones compared with other tumor zones, suggesting that even immune-hot tumors contain cells capable of immune escape. Our findings support a model whereby a subpopulation of morphologically plastic cancer cells with dysregulated DNA repair promotes ovarian cancer progression through positive selection by immune evasion.

## Abstract (web summary)

Cancer cells can actively engage in overcoming microenvironmental constraints such as tissue stiffness through adapting their shapes; however it is unclear how microenvironmental cells shape cancer nuclear morphology in human tumors in situ. Here the authors merge machine learning, digital pathology and spatial statistics to study this issue; furthermore the authors identify decreased immune infiltration in the surrounding of diversified cancer cells in a subset of ovarian tumors.

## Introduction

Plasticity of cancer nuclear shape is essential for cells to assume a variety of critical functions such as migration and metastasis^1,2^. Both external signals from the microenvironment and internal changes in the nuclear envelope components can trigger alterations in nucleus shape^2–4^, and deregulate important pathways such as DNA repair^5–7^. Conversely, cancer cells can actively engage in overcoming microenvironmental constraints such as tissue stiffness^8,9^ by adapting their shapes. Although nuclear shape irregularity is routinely assessed in diagnostic histology and the nuclear envelope is under intense investigation, little is known about the microenvironmental signals that shape cancer nuclear morphology in human tumors in situ. This is largely due to difficulties in reproducing complex human tumor microenvironments in vitro, and a lack of quantitative large-scale data on cancer morphology and spatial variability of the human tumor microenvironment.

Changes in expression of nuclear envelope components including Lamin A/C^10–12^, Emerin^13^, and *NUP88*^14^ have been identified in ovarian cancer. High-grade serous ovarian cancer (HGSOC) is the most common histological subtype of ovarian cancer, with a staggeringly low five-year survival rate of 30–40%^15,16^. Previously, we proposed a HGSOC subtyping method based on the abundance of stromal cells and lymphocytes in histology samples^17^. Genomic analysis also revealed prognostic molecular subtypes with distinct microenvironmental features such as the Immunoreactive subtype^18,19^. However, influences from the microenvironment should be regional, dictated by the high spatial heterogeneity in solid tumors^20–22^. Conventional histology and genomic analysis typically do not reveal spatially explicit information about the microenvironment. Therefore, how regional differences in microenvironmental selective pressures actively shape cancer nuclear morphology has not been studied. Unbiased, large-scale analysis of cancer morphology within the spatial context of the local microenvironment has the potential to generate more powerful predictive models and identify new targets for this aggressive cancer type. Such studies can offer new understanding into the adaptive advantage of cancer morphological plasticity, akin to understanding the morphological diversity of a species across geographical locations in ecology.

In this paper, we developed a new way of studying how microenvironmental niches shape cancer nuclear morphology by combining machine learning, digital pathology and spatial statistics. Integration of morphological and molecular data in 514 HGSOC tumors: 1) led to the identification of cancer morphological diversification as a spatial measure implicated in deregulation of DNA repair, loss of nuclear integrity and increased disease mortality; 2) provided empirical evidence that a subpopulation of cancer cells with morphological diversification could possess a selective advantage in locally immunosuppressive microenvironments; 3) supports a model of morphological plasticity as a tumor ecological process with profound clinical implications.

## Results

### Spatial mapping of morphological diversification

**Fig. 1:**
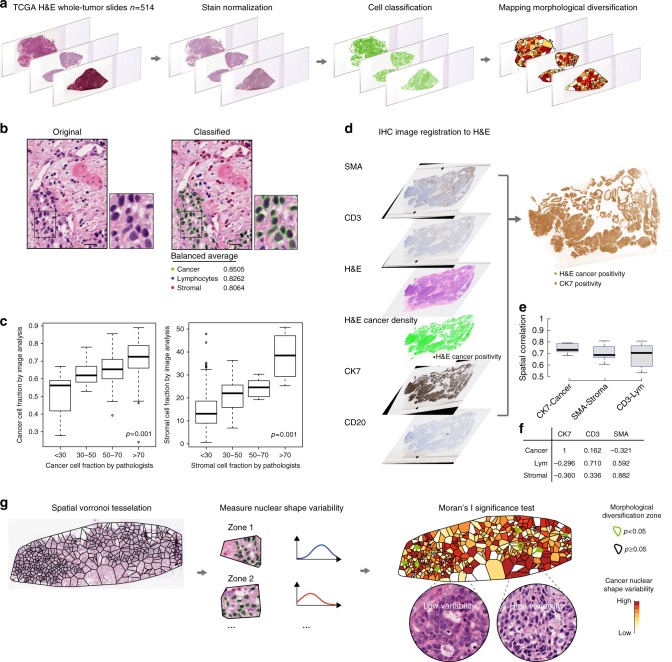
Our computational pipeline for the identification of cancer nuclear morphological diversification zones. **a** H&E-stained whole-tumor section slides were digitized and stain normalized. Single cells were classified based on their morphology. Voronoi tessellation was employed to subdivide the tumors into polygons. Morphologically diversified zones were detected using local Moran’s I statistics. **b** An illustrative example of single-cell classification: cancer cells (green), stromal cells (red), and lymphocytes (blue). Nucleus boundaries were generated by automated image analysis. Accuracy was assessed using balanced average, which is the mean of sensitivity and specificity. Scale bar shows 20 μm. **c** Jonckheere trend test of automated versus pathologist’s cell abundance scoring of cancer cells and stromal cells as a second method for validation. Boxplot center line, bounds of box and whiskers represent here and henceforth median, inter-quartile range and extreme values (1.5 times inter-quartile range). **d** Our image registration pipeline for validating H&E image analysis using serial IHC sections. An example of overlaying H&E-based cancer identification result (green points showing cancer-positive regions) on CK7 was shown. **e** Boxplot to show the spatial correlation between CK7 and H&E-based estimate of cancer cells, CD3 and lymphocytes, and SMA and stromal cells in all IHC validation samples. **f** Spearman correlation of sample-level scores from H&E image analysis (Cancer%, Lymphocyte%, Stromal%) versus IHC CK7, CD3 and SMA scores. **g** An illustrative example of running local Moran’s I analysis: a tumor section was spatially divided using Voronoi tessellation; cancer cell nuclei in each zone analyzed with respect to shape variability; and the resulting heatmap of shape data superimposed with significance test results. Images are for illustrative purpose only and do not reflect actual size of the spatial zones. Heatmap colors represent shape variability of cancer cell morphology in the spatial zone. Spatial zones identified to be morphologically diverse were outlined in green

To enable single-cell classification of hematoxylin & eosin (H&E)-stained whole-tumor histology slides for HGSOC, we developed an image processing pipeline, building on our previous studies^17,23^ (Methods, fig. 1a). Stain normalization^24^ and automated artefact detection were implemented to account for the high levels of variability in The Cancer Genome Atlas (TCGA)^25^ images. On average, 184,541 cancer cells (standard deviation ± 152,271), 34,923 lymphocytes (±31,114) and 39,610 stromal cells (±35,443) were identified, with quantitative morphological analysis, in each whole-section sample. A total of 106,620,458 cancer and 40,559,923 microenvironmental cells was identified in the entire cohort. We subsequently tested the accuracy of our pipeline with five orthogonal data types. First, the balanced accuracy as an average of specification and sensitivity of our classifier was 80.6% for stromal cells, 85.0% for cancer cells, and 82.6% for lymphocytes (fig. 1b, Methods). Secondly, automated cell scoring using image analysis was highly correlated with the independent scoring provided by TCGA pathologists (fig. 1c) (lehirdly, using gene expression data and enrichment analysis^26–28^, we identified significant associations between cell abundance and relevant functional pathways and biological processes including cell cycle and checkpoints for cancer cells, chemokine and leukocyte transendothelial migration for lymphocytes, and matrisome and collagen formation for stromal cells (Supplementary Table 1), supporting the biological relevance of the image analysis results. Next, tumor purity measures from gene expression-based method ESTIMATE and copy number-based ABSOLUTE correlated with tumor cellularity estimated by image analysis (ESTIMATE Spearman’s rho = 0.44, *p* = 0; ABSOLUTE, rho = 0.43, *p* = 0). These correlations were higher than the correlations between molecular measures and pathologists’ scores (Spearman’s rho = 0.31 for ABSOLUTE and pathologist, rho = 0.35 for ESTIMATE and pathologist). Finally, high spatial and sample-level correlations between H&E-based estimates and immunohistochemistry (IHC) sections of cancer, lymphocyte, and stromal markers on a validation set further demonstrated the validity of automated H&E image analysis (Fig. 1d–f, Supplementary Data 1, Supplementary Figure 1 and Methods).

Based on the morphological features and spatial distribution of cancer cells, we used spatial tessellation to partition tumors into non-overlapping zones, followed by a spatial statistical significance test to identify zones where cancer nuclei diversified in shape (Methods, Fig. 1g). On average, there were 20 diversification zones (±8) per tumor when diversification was present, and a diversification zone contains 350 (±194) cancer cells. 53.3% of the tumors (276/514) presented at least one morphological diversification zone.

### Disruption of nuclear envelope integrity in diversified tumors

**Fig. 2:**
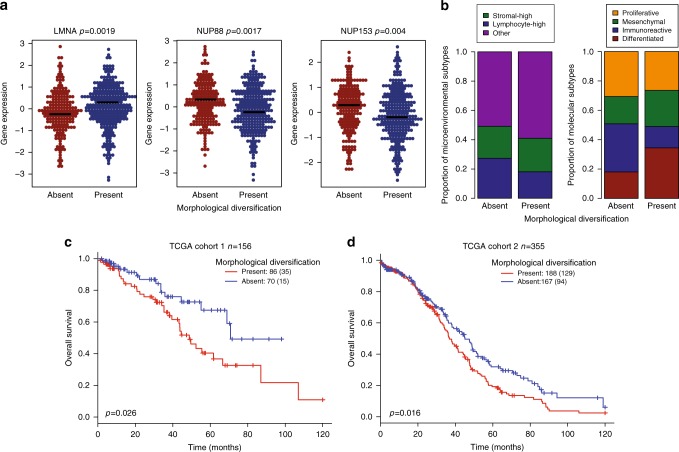
The biological and clinical relevance of morphological diversification. **a** Swarm plots for *LMNA*, *NUP88*, *NUP153* expression in samples with and without diversification. **b** Barplots illustrating the fraction of microenvironmental subtypes (left) and molecular subtypes (right) stratified by diversification. **c** Kaplan-Meier curves to show the prognostic value of diversification in OS for TCGA cohort 1 and **d** cohort 2

We first tested if known relevant nuclear envelope components in ovarian cancer were deregulated according to cancer nuclear morphological diversification, including *LMNA*, Lamin B1 and B2, Emerin, nucleoporins *NUP88* and *NUP153*^2^. The presence of morphological diversification zone was associated with increased expression of *LMNA* and decreased expression of *NUP88* and *NUP153* (Fig. 2a), although not with *LMNB1*/2 or Emerin expression (*p* > 0.05, Kruskal–Wallis test). These associations remained significant in whole transcriptome differential expression analysis after multiple testing corrections (Supplementary Data 2). However, within the diversified patient group, the fraction of diversified zones among all zones did not further correlate with the expression level of these genes (*p* > 0.05). In addition, tumors presented with at least one morphological diversification zone were less likely to be immune-high tumors based on microenvironmental composition from histology image analysis (Fig. 2b, Supplementary Figure 2). Consistent with this, less Immunoreactive (*p* = 5.37 × 10^-6^, Fisher’s test) tumors were found to present morphological diversification (Fig. 2b). On the other hand, our classification of morphological diversification presence was not influenced by tumor size, debulking status, and tumor cellularity (Supplementary Figure 3).

We then sought to determine the clinical implication of morphological diversification in HGSOC. In both TCGA cohorts defined by contributing sites, presence of morphological diversification was associated with poor overall survival (OS) but not relapse-free survival (RFS) (Fig. 2c–d, OS Cohort 1: *n* = 156, *p* = 0.026, HR = 1.99 [1.08–3.66]; Cohort 2: *n* = 355, *p* = 0.016, HR = 1.39 [1.06–1.81]). In comparison, morphological measures that were not spatially explicit were not prognostic (mean, median and standard deviation of the shape feature: all *p* > 0.1, log-rank test), highlighting the importance of studying the spatial variability in cancer morphology. Applying the same analysis to lymphocytes and stromal cells showed no diversification for lymphocytes and less frequent (19%) diversification for stromal cells with no correlation with prognosis, indicating that diversification was predominately a clinically relevant aspect of cancer cell biology. Multivariate analysis further demonstrated the independent prognostic value of diversification presence, after adjusting for clinical variables including debulking status, age, and microenvironmental features (Table 1). Importantly, the fraction of diversified zones among all zones did not further stratify the diversified patient group (*p* > 0.05, log-rank test). Therefore, the presence, independently on the quantity, of morphologically diverse cancer cells could indicate clinically relevant intra-tumor heterogeneity. We henceforth focused on the binary classification of morphological diversification based on the presence instead of quantity of diversification zones in this study.

**Table 1:** Prognostic value of morphological diversification in ovarian cancer using disease-free and overall survival. Only variables found to be significant in univariate analysis were included in multivariate analysis

Type	Variable	RFS 10 year (*n* = 440)			OS 10 year (*n* = 511)		
		HR (CI)	*p*	Conc	HR (CI)	*p*	Conc
Uni	Debulking	0.75 (0.58–0.98)	0.031*	0.524	0.74 (0.57–0.96)	0.021*	_0.543_
Uni	Age	1.01 (0.99–1.02)	0.327	0.531	1.02 (1.01–1.03)	0.0004**	0.599
Uni	Stage	0.88 (0.52–0.42)	0.266	0.537	1.07 (0.48–0.34)	0.34	0.555
Uni	Stromal-high	1.50 (1.13–2.00)	0.005*	0.515	1.22 (0.90–1.64)	0.203	0.515
Uni	Lymphocyte-high	0.74 (0.55–0.99)	0.041*	0.521	0.66 (0.48–0.91)	0.01*	0.535
Uni	Immunoreactive	0.67 (0.5–0.92)	0.013*	0.535	0.7 (0.5–0.96)	0.035*	0.518
Uni	Diversification	1.28 (1.01–1.61)	0.038*	0.518	1.45 (1.14–1.85)	0.0022*	0.538
Multi	Debulking	0.73 (0.56–0.95)	0.027*	0.557	0.77 (0.59–1.02)	0.064	0.622
	Age	-	**-**		1.02 (1.01–1.03)	0.0003**	
	Stromal-high	1.21 (0.89–1.64)	0.22		–	_**–**_	
	Lymphocyte-high	0.74 (0.53–1.04)	0.08		0.70 (0.50–0.97)	0.034*	
	Diversification	1.17 (0.92–1.51)	0.193		1.32 (1.02–1.71)	0.03*	

Diversification, patients with at least one diversification zone; Uni, univariate Cox regression; Multi, multivariate Cox regression; Conc, concordance; HR, hazard ratio; CI, confidence interval

*p  < 0.05; **p   < 0.01

### Concordant deregulation of DNA repair in diversified tumors

**Fig. 3:**
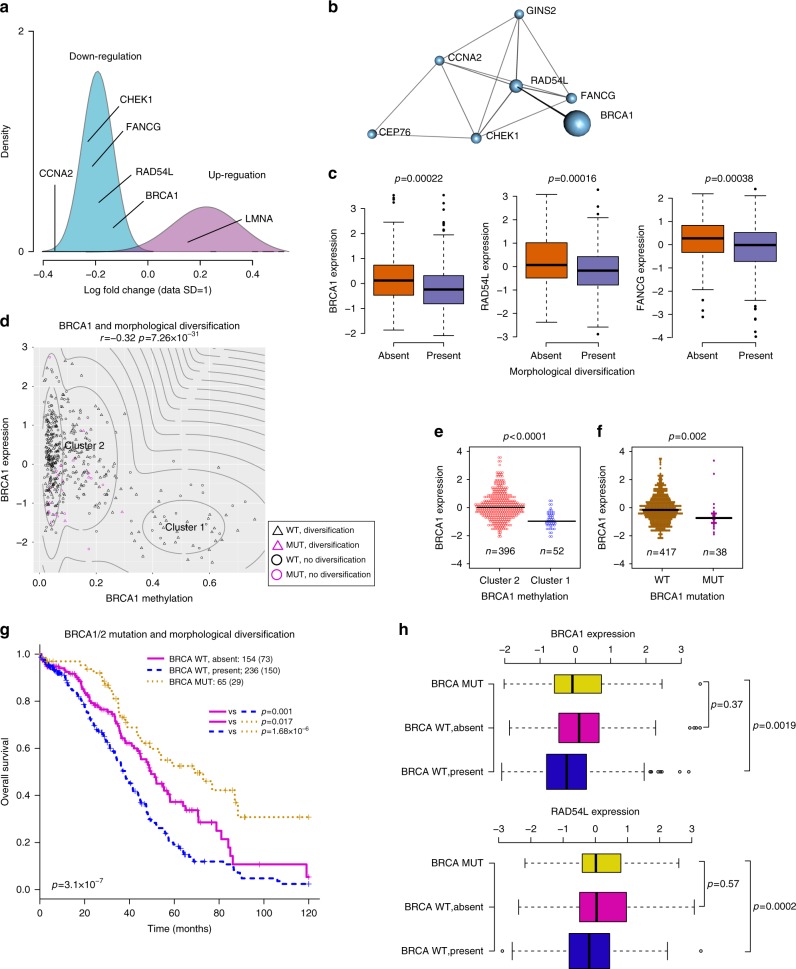
Cancer morphological diversification and DNA repair. **a** Density plot showing the distribution of log-fold change in gene expression that significantly differed between diversified and non-diversified samples. Two clusters were identified using Gaussian mixture models, which represented down- and up- regulated gene clusters. **b** The gene module formed by highly co-expressed genes (cor > 0.38, *p* < 0.001) within the top 10 genes in the differential expression analysis. **c** Boxplots to illustrate the difference in *BRCA1*, *RAD54L* and *FANCG* expression in samples with and without diversification. **d** Scatterplot illustrating the distribution of *BRCA1* expression according to *BRCA1* methylation status with contours from unsupervised clustering identifying the *BRCA1* epigenetically silenced group (Cluster 1). **e** Beeswarm plot to show the distribution of *BRCA1* expression in the two clusters shown in **d**. **f** Beeswarm plot to show significantly lower expression of *BRCA1* in a small number of samples with *BRCA1* mutation (germline and somatic). **g** Kaplan-Meier curves depicting the differences in OS for patients stratified by *BRCA1/2* mutation status (wildtype WT or mutated MUT) and morphological diversification. **h** Boxplots showing the differences in *BRCA1* and *RAD54L* expression according to the three patient groups in G

To decipher the molecular basis of morphological diversification, we examined transcriptional, copy-number, mutation and methylation profiles in TCGA. A concordant down-regulation of key homologous recombination DNA repair genes in diversified samples was evident. Among the top 10 genes ranked by differential expression analysis, *RAD54L*, *FANCG*, and *BRCA1* formed a co-expression network module together with DNA damage checkpoint *CHEK1*, cell cycle gene *CCNA2, GINS2* in DNA replication and centrosomal protein *CEP76* (Fig. 3a–c, Supplementary Data 2). Enrichment analysis revealed cell cycle and DNA repair Gene Ontology terms in down-regulated genes for diversified samples (Supplementary Data 3). Although copy number or methylation data alone did not add further information on DNA repair (Supplementary Data 4–5), their integration with gene expression revealed strong *cis*-driven effects from copy number alterations (*p* < 0.0001, ANOVA) but not methylation (*p* > 0.05, ANOVA) on the down-regulation of *RAD54L* and *FANCG* (Supplementary Figure 4). Among all 33 known oncogenic and suppressive drivers reported in HGSOC^29^, only *BRCA1* expression was associated with cancer morphological diversification. In concordance with a previous report^18^, *BRCA1* expression was lower in the epigenetically silenced group and *BRCA1* mutated samples compared with the non-silenced and wildtype group, respectively (Fig. 3d–f). However, diversified samples were not enriched for mutations in any gene or *BRCA1* methylation (*p* > 0.05). This suggests that diversification is a morphological marker of DNA repair dysregulation.

Indeed, morphological diversification could be used to further stratify *BRCA1*-WT patients for OS and RFS (OS: *p* = 0.0016, HR = 1.55 [1.18–2.04], RFS: *p* = 0.017, HR = 1.38 [1.06–1.80]), but not the *BRCA1*-mutated group (*p* > 0.05, log-rank test, Supplementary Figure 5. By merging *BRCA1* and *BRCA2* mutation status, we identified three major patient groups with distinctly different prognosis: the *BRCA1/2* mutated group with the best prognosis (*BRCA1/2*-MUT compared with all other samples: OS *p* = 2.82 × 10^−5^, HR = 0.44 [0.30–0.66]), the *BRCA1*/*2*-WT and diversification group with the worst prognosis (OS *p* = 5.264 × 10^−^^7^, HR = 1.83 [1.44–233]), and the intermediate *BRCA1/2*-WT without diversification (e. *BRCA1* and *RAD54L* expression were significantly lower in the *BRCA1/2*-WT, diversified group than in the BRCA1/2-WT not-diversified group and even the *BRCA1/2*-MUT group (Fig. 3h). Taken together, these data emphasize the specificity of DNA repair dysregulation as a major oncogenic process underlying morphological diversification.

### Unifying tumor microenvironment, cancer morphology, and genetics

**Fig. 4:**
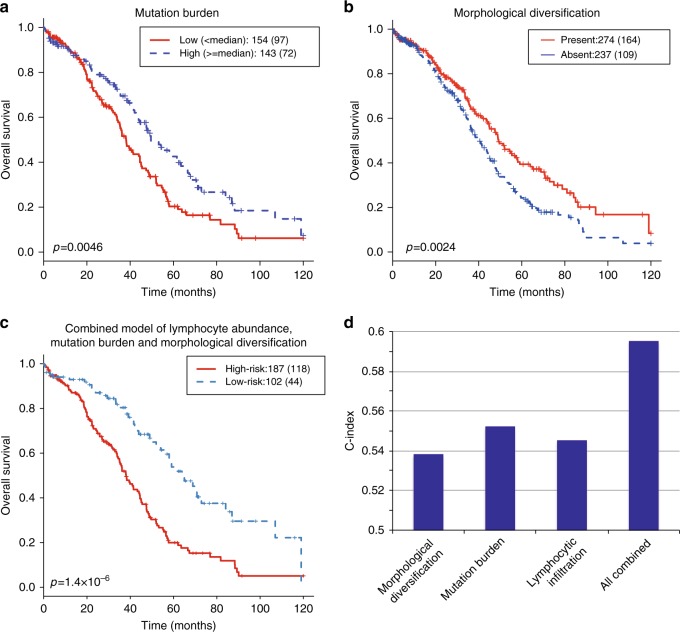
Integrated model of mutation burden, lymphocyte abundance and morphological diversification. **a** Kaplan-Meier curves to show the difference in OS according to mutation burden**. b** Kaplan-Meier curves to show the difference in OS according to morphological diversification. **c** Kaplan-Meier curves for the combined model consisting of lymphocytic abundance, mutation burden and morphological diversification. High-risk group included the patients with low mutation burden and lymphocyte abundance, but when they were in disagreement (one high and the other low), morphological diversification was present. **d** Barplot showing the model concordance (C-index) for individual measures and combined

Because morphological diversity has been associated with genetic intra-tumor heterogeneity^30,31^, we investigated the association between tumor morphological diversification and mutation burden (*n* = 297). Mutation burden was not significantly associated with diversification (*p* = 0.079, Supplementary Figure 6A). However, high mutation burden was associated with favorable OS and RFS (OS: *p* = 0.003, HR = 0.63 [0.47–0.86], RFS: *p* = 0.04, HR = 0.64 [0.47–0.87], Fig. 4a), which may be explained by an increase of lymphocytic infiltration in these samples (*p* = 0.003, Kruskal–Wallis test, Supplementary Figure 6B). While mutation burden, lymphocytic infiltration, and diversification each held strong prognostic value, they independently contributed to a combined model that was highly prognostic (Fig. 4b–d, Supplementary Figure 7). This highlights an opportunity for developing a clinical test to identify high-risk patients by combining knowledge of tumor microenvironment, cancer morphology and genetics.

### Spatial interplay with lymphocytic infiltration

**Fig. 5:**
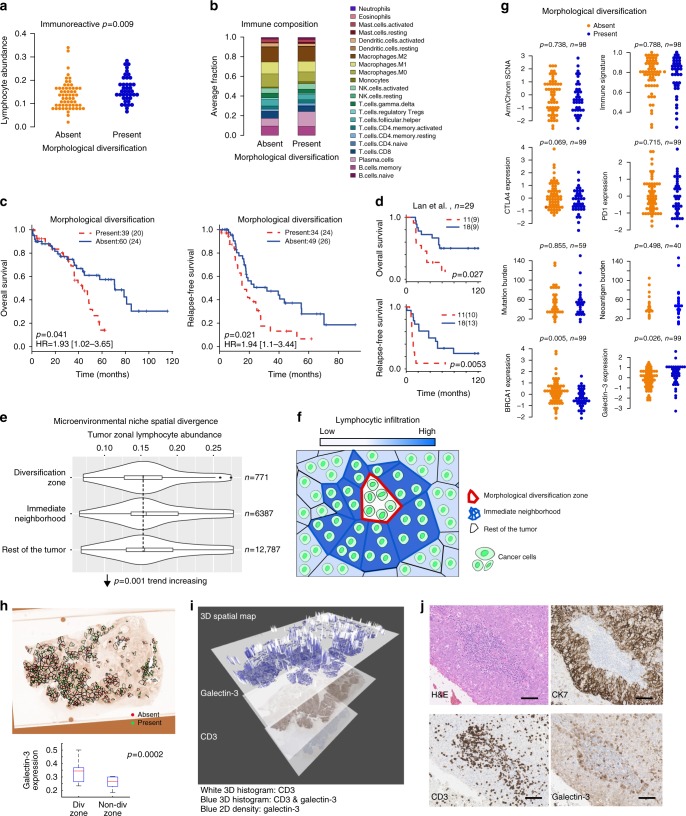
Subtype-specific analysis of morphological diversification identifies potential immune evasion in the Immunoreactive subtype. **a** Swarmplot to illustrate the difference in lymphocyte abundance according to morphological diversification within the Immunoreactive subtype. **b** Difference in immune composition according to diversification using CIBERSORT. Proportions of naïve B cells were small and therefore invisible. **c** Kaplan-Meier curves to illustrate the difference in OS and RFS according to diversification in Immunoreactive patients. **d** Kaplan-Meier curves to illustrate the difference in OS and RFS according to diversification in a validation set of 29 HGSOC with high lymphocytic infiltration. Color scheme follows **c**. **e** Violin plot showing a visually subtle spatial trend of decreased lymphocytic infiltration in diversified zones compared with their immediate neighborhood and the rest of the tumor. **f** Schematic drawing illustrating decreased lymphocytic infiltration in the diversification zone, despite an accumulation of lymphocytes in the immediate neighborhood. **g** Differences in Arm/Chrom SCNA, cytotoxic immune signature score, *CTLA* and *PD1* expression, mutation burden, neoantigen burden, *BRCA1* and galectin-3 gene expression in samples with/without diversification. **h** An illustrative example of spatial correlation analysis of galectin-3 expression and morphological diversification. Spatial tessellations from H&E morphological diversification analysis were superimposed onto a galectin-3 IHC image of a serial slide. Only zones containing galectin-3 positive cells were shown for illustrative purpose. Red points denote zones without diversification, and green points denote zones with diversification. Boxplot shows the difference in galectin-3 expression between all diversified and non-diversified zones across validation samples. **i** 3D spatial map to illustrate the spatial relationship between galectin-3 and CD3 + cells in a sample. **j** An illustrative example of galectin-3 expression at the cancer-lymphocyte interface. Scale bar shows 30μm

To further investigate the interplay between cancer morphological plasticity and the microenvironment, we focused on the Immunoreactive subtype with the assumption that, in general, strong microenvironmental influence existed in this subtype. Interestingly, only within this subtype, diversification was associated with increased lymphocyte abundance (Fig. 5a, Supplementary Figure 8). Consistent with this, CIBERSORT^32^ analysis using gene expression data revealed increased plasma cells (*p* = 0.022, Kruskal–Wallis test) and, in smaller proportions, naïve B cells (*p* = 0.034, Kruskal–Wallis test) in morphologically diversified samples (Methods, Fig. 5b). Despite having an immune-hot microenvironment, morphologically diverse tumors were more aggressive, with 14.5% OS and 6.7% RFS at year 5 compared with 53.3% OS and 31.1% RFS for tumors without diversification (Fig. 5c). The association between morphological diversification and poor prognosis was reproducible in an independent validation set of HGSOC with high lymphocytic infiltration (*n* = 29, Fig. 5d). In contrast, none of the clinical variables tested, including debulking, age and stage, as well as lymphocyte abundance, was associated with OS or RFS within this subtype (Table 2).

**Table 2:** Prognostic value of morphological diversification in the Immunoreactive subtype

Type	Variable	RFS 10 year			OS 10 year		
		HR (CI)	*p*	Conc	HR (CI)	*p*	Conc
Uni	Diversification	1.94 (1.1–3.44)	0.023*	0.575	1.93 (1.02–3.65)	0.044*	0.528
Uni	Debulking	1.01 (0.54–1.92)	0.97	0.498	0.71 (0.37–1.36)	0.31	0.578
Uni	Age	0.98 (0.96–1.01)	0.227	0.501	1.02 (0.99–1.05)	0.073	0.604
Uni	Stage	1.22 (0.44–3.41)	0.707	0.512	1.77 (0.74–4.22)	0.189	0.534
Uni	Lymphocyte abundance	0.86 (0.49–1.5)	0.588	0.52	0.85 (0.47–1.54)	0.596	0.561

* *p* < 0.05

To further interrogate the relationship between immune ecology and cancer morphological diversification, we subsequently performed spatial analysis within each tumor, and identified a negative spatial correlation between morphological diversification and zonal lymphocyte abundance that was visually subtle but statistically significant (*p* = 0.001, Jonckheere trend test, Fig. 5e–f). Therefore, we speculated that diversifying cancer cells evolved immune evasion to overcome the high level of immune selective pressure in this subtype. We first sought to identify molecular features that could inform the underlying biology. However, diversification was not associated with Arm/Chrom somatic copy number alteration (SCNA), cytotoxic immune signature, mutation burden, *CTLA4*/*PD1* expression, or predicted neoantigen burden (Fig. 5g, Supplementary Data 6 and Methods). Nevertheless, only in the Immunoreactive but not any other subtypes, diversified samples had significantly higher expression of galectin-3, which inversely correlated with *BRCA1* expression (Fig. 5g, Supplementary Data 2). Since galectin-3 has been previously implicated in immune evasion by inducing T cell apoptosis^33,34^, we examined its expression within the immune contexture in a validation sample set. This was achieved by imposing spatial tessellations from H&E morphological diversification analysis onto galectin-3 IHC images, which enabled direct spatial analysis to test the relationship between diversification and lymphocyte abundance as spatial variables (Fig. 5h, Methods). Overall, a negative spatial relationship between galectin-3 expression on cancer cells and CD3 + cells was identified (mean Spearman’s rho = −0.40). In addition, we observed galectin-3 expression at the interface between cancer and lymphocyte aggregates (Fig. 5i–j, Methods, Supplementary Data 1). These preliminary data support the role of galectin-3 as a potential immune suppressor in HGSOC, which warrants further investigation.

## Discussion

In this study, we investigated the spatial heterogeneity of cancer cell nuclear morphology in HGSOC. This led to the identification of tumor molecular, spatial and ecological forces that could influence morphological diversification over space. Understanding morphological diversity within the spatial context of ecological environment was fundamental to the discovery of Darwinian evolution. However, studying complex morphology of cancer cells within the tumor microenvironment is particularly challenging, due to the high microscopic complexity in human tumors and limitations in model systems in representing this complexity. By developing a systems approach to define morphological diversification of cancer nucleus, we demonstrated that the presence of morphologically diverse cancer cells predicted poor overall survival, an observation that cannot be explained by known clinical and genetic factors in HGSOC. About half of the samples contained diversified zones, and within this subset, increased number of such zone did not further correlate with survival, suggesting that risk did not increase with abundance but pertained to the presence of morphologically diverse cells. This is in line with the paradigm that cancer evolution is often driven by rare but significant events, for example minor subclones that drive progression and resistance^35^. In addition, when morphological data were averaged for all nuclei within the tumor, no prognostic value was found, underscoring the importance of examining the spatial heterogeneity but not tumor average of cancer morphology.

Morphological diversification was associated with dysregulation of *LMNA*, *NUP88*, and *NUP153*, which are key genes in maintaining nuclear envelope architecture and structural integrity^2,36–38^, but not Lamin B1/B2. This may be explained by the specificity of the nuclear pore protein *NUP88* in binding the tail domain of *LMNA* but not of Lamin B1/B2^39^. The data points towards the disruption of *NUP88* functions in diversified samples, which can induce aneuploidy formation and tumorigenesis^40^. In addition, depletion of *NUP153* can result in impaired post‐mitotic assembly of the *LMNA*, leading to a polymorphic, lobular nuclear shape^36,37^. Therefore, these molecular data support the biological relevance of morphological diversification.

Furthermore, our findings highlight morphological diversification as a *BRCA1*-related process of disease progression in HGSOC. Concordant down-regulation of key DNA repair genes was identified in diversified samples. *RAD54L, FANCG*, *BRCA1*, and DNA damage checkpoint *CHEK1* were among the most significant genes in differential expression analysis and formed a co-expression gene module. *FANCG* is one of the six genes associated with Fanconi anemia that governed the Fanconi anemia-*BRCA* (*FANC*-*BRCA*) pathway together with *BRCA1*/2^41^. Disruption of this pathway can induce cisplatin resistance^41^, providing a potential explanation for the poor prognosis of morphologically diverse samples. Paradoxically, deficiency of *RAD54L*, *CHEK1*, and *BRCA1* is known to induce sensitivity to PARP inhibition and correlate with good prognosis, due to their involvement in the repair of double-strand breaks by homologous recombination^42,43^. However, *BRCA-*WT patients with diversification had significantly lower *BRCA1* and *RAD54L* expression and worse overall survival, even when compared to the *BRCA*-MUT group. Therefore, although the association between *BRCA1* and tumor morphology has been previously reported^44^, morphological diversification represents a novel subtype with dysregulated DNA repair functions and is urgently in need of new treatment strategies.

In addition, our proposed morphological features of HGSOC, identified using fully automated tumor spatial analysis, provided independent prognostic value to existing prognostic features of HGSOC such as lymphocytic infiltration and mutation burden. Together, these results support the development of a new clinical test to identify high-risk patients by building on the biological relevance and interactions among the tumor microenvironment, cancer morphology and genetics. We postulated that evasion of immune recognition could be responsible for the poor prognosis of morphologically diverse tumors. In the Immunoreactive subtype, morphological diversification correlated with poor prognosis, despite an increase of lymphocytes including plasma and naïve B cells that were favorable prognosticators in HGSOC^45–47^. While none of the clinical variables, SCNA, mutation burden or checkpoint expression can explain this difference in prognosis, we uncovered a negative spatial relationship between diversifying cancer cells and immune infiltration, indicating immune evasion in localized tumor zones. This was achieved by examining spatial association between morphological diversification and lymphocytes within each tumor. Hence, we demonstrated that spatial analysis performed in human tumors, based on the emerging concept, space as a surrogate, can be a useful tool for maximizing inference about ecological processes^20^.

Increased expression of galectin-3 in Immunoreactive samples supports a speculative theory of immune evasion for diversifying cells. We uncovered preliminary evidence that indicates a role of galectin-3 in immunosuppression in HGSOC, in line with its known function in inducing T cell apoptosis in melanoma^34^. Our quantitative data, while limited by access to small validation cohorts, demonstrated a negative spatial relationship between galectin-3 expression and CD3+ cell infiltration. These results call for further investigations to elucidate the mechanism by which diversifying ovarian cancer cells mediate and influence immune escape. Taken together, our data support a model in which a subpopulation of cancer cells, morphologically diversified in shape, is capable of immune evasion within an immune-hot tumor. Such cancer cells may co-evolve within a locally immunosuppressive tumor microenvironment, bypassing immune surveillance and promoting disease progression as a consequence. Identifying factors driving immune escape in these tumors will lead to improved understanding in immunosuppression and advances in immunotherapy for maximum therapeutic gain in HGSOC.

## Methods

### Patient selection

This study included 514 patients with International Federation of Gynecology and Obstetrics (FIGO) stage II-IV HGSOC from TCGA, for whom H&E-stained whole-tumor sections from treatment-naïve tumor specimens were available. All specimens were obtained with consent from the relevant institutional review board participated in TCGA. Samples were split into a discovery cohort that included the two biggest centers (University of Pittsburgh and Memorial Sloan Kettering, *n* = 159) and a validation cohort (remaining centers, *n* = 355). After surgery, all patients received a platinum agent and 94% of patients also received a taxane. Clinical parameters included OS and RFS, age, recurrence, FIGO stage, debulking and platinum sensitivity status (Table 3). OS was censored at the date of death or, for living patients, the date of last contact. RFS was defined as the interval from the date of initial surgical resection to the date of progression, date of recurrence, or date of last known contact if the patient was alive and had not recurred. For validating survival analysis in the Immunoreactive subtype, a set of HGSOC samples (*n* = 29) with prominent immune infiltration was used^17^. For IHC experiments, a second validation set of treatment naïve, stage-matched samples (*n* = 7) was obtained with appropriate ethical approval under the Royal Marsden Hospital NHS Foundation Trust study CCR3705 (Supplementary Data 1).

**Table 3:** Patient clinicopathologic characteristics stratified by presence/absence of morphological diversification

	Morphological diversification		
Factor	Present	Absent	*P*
Number	276	238	
Analyzed subimages	57109	84322	
Cancer cells	55,059,416	51,561,042	
Lymphocytes	10,303,767	8,979,007	
Stromal cells	12,266,456	9,010,693	
*Age*			0.91
Median	58	59	
Range	(36–87)	(26–87)	
*Death*			0.003*
No	132 (47.8%)	92 (38.7%)	
Yes	144 (52.2%)	145 (60.9%)	
Unknown	0 (0%)	1 (0.4%)	
*Recurrence*			0.002*
Yes	163 (59.1%)	126 (52.9%)	
No	2 (0.07%)	12 (5%)	
Unknown	111 (40.2%)	100 (42%)	
*Molecular subtype*			1.3 × 10^−06^*
Differentiated	95 (34.4%)	33 (13.9%)	
Immunoreactive	40 (14.5%)	60 (25.2%)	
Mesenchymal	68 (24.6%)	34 (14.3%)	
Proliferative	73 (26.4%)	56 (23.5%)	
Unknown	0 (0%)	55 (23.1%)	
*FIGO stage*			0.018
IIa	1 (0.4%)	2 (0.8%)	
IIb	3 (1.1%)	1 (0.4%)	
IIc	6 (2.2%)	13 (5.5%)	
IIIa	6 (2.%)	0 (0%)	
IIIb	10 (3.6%)	13 (5.5%)	
IIIc	199 (72.1%)	180 (75.6%)	
IV	48 (17.4%)	28 (11.8%)	
Unknown	3 (1.1%)	1 (0.4%)	
*Debulking*			0.38
Optimal	170 (61.6%)	159 (66.8%)	
Suboptimal	74 (26.8%)	57 (23.9%)	
Unknown	33 (11.6%)	22 (9.2%)	
*Platinum status*			0.23
Resistant	55 (19.9%	28 (11.8%)	
Sensitive	110 (39.9%)	79 (33.2%)	
Unknown	111 (40.2%)	131 (55%)	

*p < 0.05

### IHC staining

All staining was performed on the Leica Bond III platform using Leica Bond Polymer Refine Detection (Leica, DS9800). Blocking of endogenous peroxidase and non-specific staining was performed as per kit instructions. Following on-board dewax, and epitope retrieval (HIER) where necessary, primary antibody was applied for 15 min, followed by rabbit anti-mouse post-primary and anti-rabbit polymer for 8 min each, all at ambient temperature. Epitope retrieval was performed on-board at 99 C using either Leica Epitope Retrieval Solution 1 or 2 (Leica, AR9961, AR9640; low and high pH solutions respectively), for the times shown below.

Protocols for individual antibodies were as follows: **CK7** (Leica, mouse clone RN7, cat. PA0138) used as supplied (ready-to-use reagent, with no further dilution), HIER with ER2 for 20 min; **CD3** (Leica, mouse clone LN10, cat. NCL-L-CD3-565), used at a dilution of 1/100, HIER with ER2 for 20 min; **CD8** (Leica, mouse 4B11, cat. PA0183) used as supplied, HIER with ER2, 20 min; **CD20** (Agilent Technologies, mouse L26, cat. M075501-2) used at 1/100, HIER with ER1, 20 min; **SMA** (Leica, mouse sm-1, cat. PA0943) used as supplied, no epitope retrieval required; **Galectin-3** (Leica, mouse 9C4, cat. PA0238), used as supplied, HIER with ER2, 20 min.

### H&E image analysis

514 H&E whole-tumor section images were subjected to fully automated image analysis for single-cell classification at a resolution of 0.5 microns per pixel. Each sample was split into non-overlapping tiles with a size 2000 × 2000 pixels using *bfconvert* from the open microscopy environment^48^. Stain normalization was performed using a nonlinear mapping approach^24^ to accommodate the high staining variability in the samples resulting from variations in tissue preparation and stain reactivity. Single cell detection and classification were performed using open source R package CRImage^23^ with an ovarian cancer cell classifier^17,49^. In brief, watershed segmentation for hematoxylin positive nuclei was performed for cell detection, and classification was based on a support vector machine^50^ with 97 morphological and textural features. Validation of the automated cell identification was performed using *n* = 894 single cell annotations (stromal cells *n* = 209, cancer cells *n* = 501 and lymphocytes *n* = 184) from 32 images provided by a pathologist (DNR) blinded to image analysis results, pathological scoring of cancer and stromal cells, gene expression and copy-number data provided by TCGA^51^.

### Validation of H&E image analysis with serial IHC sections

We developed an image analysis pipeline to validate H&E image analysis using multiple serial IHC sections, which combined automated image registration, IHC image and spatial analysis. In brief, multiple sections were cut and placed in the same orientation on the slides, with the H&E midway through the series. The remaining sections were stained with CK7, CD3, CD8, CD20, SMA, galectin-3, respectively. These were digitalized and spatially aligned to H&E using an image registration algorithm^52^. The accuracy of image registration was evaluated (average Dice coefficient = 0.91). Results of H&E-IHC correlation were reported as sample-level cross-correlations between the two assays and within-sample spatial correlations at a spatial resolution of 64 μm (~8–10 cells) (Supplementary Data 1).

IHC image registration to H&E was performed using a two-stage approach: an initial rigid alignment followed by a non-linear refinement. The first stage of the registration was performed by aligning the external boundaries of the tissue sections^52^. The initial rigid registration was more likely to be inaccurate at high resolution, due to non-linear physical distortions that occur during sectioning. The second stage process corrected for this by performing a refinement procedure at high resolution to generate a non-linear registration transformation, based upon the initial rigid registration. The refinement was a local rigid alignment of salient tissue structures, such as nuclei clusters, and was performed on 500 × 500 pixel regions, sampled at a resolution of 0.46 microns/pixel. Coordinates of the corners of each region of interest were used as reference points to find the best-fit non-linear transformation. Here a 4th degree polynomial transformation was used. The accuracy of image registration was assessed. We used the DICE coefficient to measure the overlap of tissues after registration (Supplementary Data 1). DICE similarity coefficient is a spatial overlap index and a reproducibility validation metric, defined as twice the overlap area divided by the sum of two tissue areas. Its value ranges from 0, indicating no spatial overlap between two sets of binary results, to 1, indicating complete overlap.

All IHC sections were first mapped onto the H&E section using the transformations generated from the registration process. Stain separation^53^ was applied to the registered RGB images to extract the intensities of the hematoxylin and IHC stains. Thresholding was applied to IHC stain channel to identify regions of positivity for each marker according to controls. Tissue regions belonging to the slide background, tissue folding, staining artefacts were not considered while calculating IHC scores. Automated IHC scoring was performed on 10 × 10 regions of interest, sampled at a resolution of 6.4 microns/pixel. Regions were scored as the percentage of positive cells. In the case of galectin-3 scoring, instead of a 10 × 10 neighborhood, all the pixels that were inside the edges of a particular Voronoi tessellation were considered to calculate the positivity score. For sample-level analysis, regional scores were averaged.

We then estimated CK7, CD3, and SMA positivity and calculated their spatial correlation with cancer, lymphocyte and stroma ratios estimated from the H&Es, respectively. We observed a positive spatial correlation between CK7 and cancer ratio (average Pearson corr = 0.74 ( ± 0.03)) calculated from 7 IHC samples. Similarly, we observed a positive spatial correlation between CD3 positivity and lymphocyte (average Pearson corr = 0.67 ( ± 0.09)) calculated from 7 IHC samples. Lastly, we observed a positive spatial correlation between for SMA, positivity and stroma ratio (average Pearson corr = 0.70 ( ± 0.06)) calculated from 7 IHC samples. The lowest correlation was observed between lymphocyte and CD3, as expected, since only a subset of lymphocytes were T cells. Because the CD20 stain was largely negative across all these samples, and CD8 was mainly used to confirm its presence among CD3 + cells, we did not use them for quantitative analysis.

### Spatial analysis of tumor morphology

Spatial partitioning of tumor sections into tumor regions was achieved using Voronoi tessellation. Because Voronoi tessellation mimics naturally emerged patterns, it is therefore particularly useful for studies of ecology^54^, and we have recently demonstrated its applicability in histology analysis^20,55^. Randomly selected cancer cells were used as seeds to create polygons that contain all their closest neighbors. Let ***K*** be a set containing all cancer cells, and let (*C*_*k*_)_*k*∈***K***_ be the coordinates of a cancer cell *k*. A Voronoi region *R*_*k*_ generated by cancer cell *C*_*k*_ contains all cells *P* that are (1) not seeds and, (2) closer to *C*_*k*_ than to any other seed *C*_*j*_, *j* ≠ *k*. Let *d*(*Q*_*i*_*,Q*_*j*_) be the Euclidean distance function between two cells *Q*_*i*_ and *Q*_*j*_, then

1 $$R_k = \left\{ {x \in P{\mathrm{|}}d\left( {x,C_k} \right) \le d\left( {x,C_j} \right),\forall j \ne k} \right\}.$$

The aim of spatial tessellation is to divide tissue areas into small regions. We have previously evaluated different methods for spatial tessellation, the most common ones being square and Voronoi^56^. Here we chose to use Voronoi due to its property in mimicking naturally arising pattern and generating a more uniformly distributed number of cells per region, compared with square tessellation. We used randomly selected cancer cells as seeds, such that the number of cancer cells from the Voronoi regions follows a normal distribution. Because the morphology of cancer cells within each spatial region was the focus here, we did not specifically account for vessels or clear areas.

Informally, this means that for each cancer cell selected as a seed, the corresponding Voronoi region consists of all cells that are closer to this cancer cell (seed) than to any other seed in the tissue. The number of cancer cells *N* selected as seeds scaled non-linearly with tissue area and was computed by

2 $$N = \frac{{{\mathrm{Tissue}}\,{\mathrm{area}}^{\frac{1}{2}}}}{3},$$

resulting in an average of 527.5 zones per tumor. Equation 2 determines the number of regions and, by choosing cancer cells as seeds, also ensures that there are cancer cells within the regions. In addition, cancer-dense regions are more likely to have seeds sampled. Therefore, it helps dissect regions with densely packed cancer cells. Because the main objective of this study is to identify spatial diversification of cancer nucleus morphology, the size of region should scale with the tissue size instead of the number of cancer cells, to avoid bias towards tissue size. For example, if it scales with the number of cancer cells, for tumors with significant fibrotic content, there will be fewer regions and therefore results would skew the spatial analysis result: existing spatial diversification in cancer morphology may not get detected due to low spatial resolution.

### Statistical identification of morphological diversification zones

Diversification zone identification was carried out by computing local Moran’s I^57^, an indicator of spatial autocorrelation for a specific spatial location. Local Moran’s I was developed for the identification of spatial hotspots or outliers^57^, and has been extensively used in ecology due to several outstanding properties compared with other methods^58–60^. When applied to the shape feature data of cancer nuclei in conjunction with their spatial locations, it can identify tumor regions containing cancer nuclei with significantly different morphology compared with other regions. Local Moran’s I for a region *i* is defined as

3 $$I_i = \left( {\Upsilon _i - \overline {\Upsilon _i} } \right)\mathop {\sum}\limits_{j = 1}^m {w_{ij}\left( {\Upsilon _j - \overline {\Upsilon _i} } \right),}$$

where $\Upsilon _i$ is the variability of the shape feature for all cancer cells *C*_*i, j=1…n*_ in *R*_*i*_ derived by

4 $$\Upsilon _i = {\mathrm{SD}}\left( {\frac{{p_{C_j}}}{{2\sqrt {{\mathrm{\pi }} \ast a_{C_j}} }}} \right),\forall j \in \{ 1, \ldots ,n\}$$

where SD is the standard deviation function, *p*_*Cj*_ is the perimeter of the nucleus of the *j*th cancer cell*, a*_*Cj*_ the nuclear area of the *j*th cancer cell and *n* the total amount of cancer cells within the region *R*_*i*_. $\bar \Upsilon$ is the average of Y_*i*_,*i*∈{1..*m*} for all regions in the neighborhood of region *R*_*i*_ and *w*_*ij*_ is the spatial weight of the connection between *i* and *j*. *w*_*ij*_ is 1 if *i* is a neighbor region of *j*, and 0 otherwise. A region is a neighbor to *R*_*i*_ if they share a common edge or point. Local Moran’s I allows to perform significance test against the null hypothesis of no spatial association, that is, spatial randomness. Following Anselin^57^, the significance test was carried out by comparing the standardized Moran’ I statistics, *z*-scores,

5 $$z_i = \frac{{I_i - E\left[ {I_i} \right]}}{{\sqrt {{\mathrm{Var}}\left[ {I_i} \right]} }},$$

to the standard normal distribution, using *p* = 0.05 as a threshold for significance and adjusted with False Discovery Rate. This determined whether a zone presents significantly different cancer nuclear morphology as compared to the global mean and neighboring zones in the tumor. In Anselin^57^, Monte Carlo permutations were recommended to obtain more accurate *p*-values compared with analytical methods, as illustrated with an example study on 42 African nations. We have compared an implementation of the Hope methodology^61^ with analytically obtained *p*-values for cancer morphological diversification and found no significant difference or advantage in using Monte Carlo simulations, possibily due to the large number of observations obtained per tumor samples (on average 527.5 zones per tumor).

### Pathological inspection

50 samples were inspected by a pathologist. In some cases (roughly 40%), increased pleomorphism could be observed in most of the diversified regions. For the remaining cases there was no immediately obvious, uniform feature that differentiates these two types of regions, suggesting that image analysis can identify visually subtle features of tumor nuclear morphology.

### Molecular data analysis

ESTIMATE^62^ and ABSOLUTE^63^ tumor purity data were downloaded from^64^. ESTIMATE uses gene expression profiles of 141 immune genes and 141 stromal genes to estimate tumor purity, stromal and immune scores (based on TCGA Agilent array-based expression profiles of ovarian cancer *n* = 417 used for ovarian cancer in the original publication). ABSOLUTE uses somatic copy-number data for inferring tumor purity. Copy number (*n* = 463), gene expression (*n* = 455), methylation (*n* = 433), mutation data and mutation burden as the total number of somatic mutations (*n* = 297) were obtained from the Broad Institute and cBioportal^65,66^. Differential gene expression analysis of samples with and without diversification was performed using the R (v3.3.1) package *limma*^67^ (v3.28.21) available via Bioconductor^68^. Gene set enrichment analysis was performed using the R package *HTSanalyzeR* (v2.26.0). Multiple testing correction was performed using False Discovery Rate^69^. Immune cell subset analysis was performed using CIBERSORT^32^ and the most variable probe for each gene was selected according to standard deviation. Gene expression was then deconvoluted using the LM22 signature matrix^32^, which contains 547 genes for the identification of 22 hematopoietic cell types. Statistical significance was assessed by generating *p*-values from 200 permutations. Only the patient subset with immune abundance greater than the median immune abundance and with internal filtering step *p* ≤ 0.05 was considered for deconvolution analysis. Hypergeometric test was used for copy number and mutation data analysis to identify specific alterations or mutations enriched in morphologically diverse samples with multiple testing corrections. Only Immunoreactive subtype contained sufficient number of samples after filtering. *BRCA1* epigenetically silenced group was derived by clustering *BRCA1* expression and methylation data using Gaussian mixture models^70^ following^18^.

### Galectin-3 expression and its relationship with CD3 expression and morphological diversification

In all of these samples, we observed a negative spatial correlation between galectin-3 expression and CD3 expression. To avoid the correlation analysis being confounded by the spatial segregation of tumor and stroma, only regions with at least 30% but no more than 70% tumor were used. We further carried out spatial correlation analysis of tumor content and galectin-3 expression but found no correlation (corr = 0.019), thereby excluding the possibility that the correlation between CD3 and galectin-3 was due to stroma-tumor spatial division. Correlation analysis between galectin-3 and CD3 was carried out using all regions as such in a sample.

H&E morphological diversification analysis was performed as previously described. 2/7 samples did not contain any diversified regions. Spatial tessellation derived from this analysis was imposed onto galectin-3 IHC images after registration. For each sample with diversification present, galectin-3 expression was quantified for each region. All regions across 5/7 samples were pooled together for testing the difference in galectin-3 expression as shown in Fig. 5h. Average galectin-3 expression in diversified and non-diversified regions for each sample was listed in Supplementary Data 1.

### Survival analysis and statistical tests

Statistical analyses were performed in R. Survival analysis was performed using the Kaplan-Meier estimate and the log-rank test. Cox proportional hazards model was used for univariate and multivariate survival analysis. Effects were expressed as hazard ratios (HR) with 95% confidence intervals (CI). Kruskal–Wallis test was used to compare sets of continuous values, Fisher’s exact test for categorical variables. The abundance of stromal cells/lymphocytes was quantified as the percentage of stromal cells/lymphocytes in all cells. Microenvironmental subtypes were identified based on the percentage of lymphocytes and stromal cells for each tumor. Lymphocyte-high subtype was determined by high lymphocyte abundance ( ≥75%) and low stromal cell abundance (≤25%), such that the number of patients was similar to that of the Immunoreactive subtype. Similarly, the Stromal-high subtype was determined as samples with low lymphocyte abundance (≤25%) and high stromal cell abundance (≥75%). With continuous variables, a range of cutoffs at 30–70 percentiles at 1% interval was searched to identify cutoffs that resulted in difference in survival. Zonal difference in lymphocytic infiltration was computed using all diversification zones, their immediately adjacent neighboring zones and remaining zones, and the Jonckheere trend test was used for testing spatial trends.

### Neoantigen prediction

For 40 of the patients in the immunoreactive subtype, we could download whole exome sequencing data from the TCGA website. Reads extracted from the TCGA bam files were realigned to human build GRCh37 (hs37d5). Our protocol for annotating neoantigens is constituted of the following steps: (i) variant calling in tumor and germline samples for each patient; (ii) annotation of neopeptides generated by somatic variants; (iii) prediction of patients’ HLA-types; (iv) prediction of neoantigens using neopeptides from (ii) and HLA-types from (iii) as input. Hereafter, we describe in details the different steps.

1. Variant calling
  : we called both germline and somatic variants using a combination of MuTect2^71^ and Platypus^72^. We first ran MuTect2 (MuTect2 -R < hs37d5.fa > -I:tumor < tumor_bam_file > -I:normal < normal_bam_file > –dbsnp < dbsnp_132_b37.leftAligned.vcf > –cosmic < hg19_cosmic_v54_120711.vcf > -o < mutect_vcf_file > ) and then used MuTect2 calls as priors for a joint normal-tumor Platypus call (platypus callVariants–refFile≤hs37d5.fa > –bamFiles≤normal_bam_file, tumor_bam_file > –output≤somatic_vcf_file > –source≤mutect_vcf_file > –minPosterior = 0–getVariantsFromBAMs = 1–logFileName ≤log_file > –verbosity = 1). From the output of Platypus, we assigned germline variants as follows: they had a PASS in the FILTER column of the Platypus output, genotype quality GQ≥10, germline sample genotype different from “0/0”, germline coverage ≥10 and at least one germline variant read. If more than one alternative variant satisfied these conditions and appeared in the Platypus-assigned genotype, we considered only the one that had the highest allele frequency. We filtered out variants found in segmental duplication regions (genomicSuperDups.bed.gz). From the output of Platypus, we assigned somatic variants as follows: we excluded variants that in the corresponding germline sample had coverage lower than 10, genotype other than “0/0”, genotype quality GQ < 10 or one or more reads carrying a variant. Somatic indels were retained if they had PASS or alleleBias in the FILTER column of the Platypus output; all other somatic variants we considered (see below for a complete list) were retained if they had any among PASS, alleleBias, Q20, QD, SC, and HapScore in the FILTER column. Further, any somatic variant had to have in the tumor sample: genotype different from “./.”, coverage≥10, at least 3 reads carrying the variant. If more than one alternative variant satisfied these conditions, we considered the one that had the highest allele frequency among those that appeared in the Platypus-assigned genotype or, if none appeared in the Platypus-assigned genotype, simply the one with the highest allele frequency. Next, we filtered out somatic variants with tumor content-adjusted allele frequency <0.15. We filtered out variants found in segmental duplication regions (genomicSuperDups.bed.gz).
2. Neopeptide generation
  : for neopeptide generation, we considered the following protein sequence–modifying variants (following the VEP^73^ classification: variant_effect_predictor.pl -i < input_vcf_file > -o < output_vep_vcf_file > –cache–dir_cache < dir_cache > –port < port# > –everything–force_overwrite–vcf): missense_variant, inframe_insertion, inframe_deletion, frameshift_variant, stop_lost and stop_gained. However, variants that additionally featured one or more among splice_acceptor_variant, splice_donor_variant, start_lost or stop_retained_variant annotation terms, were discarded. As a source for protein sequences we used the file Homo_sapiens.GRCh37.75.pep.all.fa downloaded from the ENSEMBL website (http://ftp.ensembl.org/pub/release-75/fasta/homo_sapiens/pep/). Neopeptides of lengths 8–11 were generated using in-house scripts according to the following protocol. For each somatic variant (see categories above) falling in a canonical transcript (canonical transcripts annotated following VEP), we ran a sliding window on the corresponding mutated protein sequence to generate all peptides of length 8–11 spanning the variant. A number of cases deserved special attention. If one or more additional variants (somatic or germline) were found within 8–11 positions of the somatic variant under consideration, we had to decide whether or not the neopeptide generated would carry none, some or all of the additional variants. Here, to simplify things, we assumed all variants (be it somatic or germline) to be ‘in phase’ with each other and with the same zygosity. This meant that if additional variants were found within 8–11 positions of a somatic variant, the neopeptides we generated carried all the variants (both somatic and germline) that were present in the protein region they spanned. Neopeptides corresponding to somatic stop_lost and frameshift variants were generated by modifying the cDNA sequence of the canonical transcript (as found in http://ftp.ensembl.org/pub/release-75/fasta/homo_sapiens/cdna/Homo_sapiens.GRCh37.75.cdna.all.fa.gz) according to the observed variant and generating the frame-shifted protein sequence until a stop codon was found or the end of the cDNA sequence was reached. We manually checked for cases in which 3_prime_UTR variants could affect the resulting neopeptides. Further, cases of frameshift and stop_lost variants where additional variants were present within the same transcript (e.g. a second frameshift variant) as well as cases where two or more neighboring variants overlapped (i.e. they affected at least one identical amino acid position) were manually inspected to determine the frame-shifted neopeptides that had to be generated. Finally, neopeptides generated by somatic mutations in a protein were excluded if they were found elsewhere in the germline version of the same protein (modified taking into account, if present, any germline missense mutation and inframe indel; if two or more germline variants were found within 8–11 positions of one another, we considered as germline peptides the ones carrying all the germline mutations that were present in the protein region they spanned). The in-house python scripts we used to generate neopeptides are available upon request.
3. HLA types
  : HLA-A, HLA-B and HLA-C types were predicted using the program Polysolver^74^ run on normal samples (shell_call_hla_type < normal_bam_file > Unknown 1 hg19 STDFQ 0 < output_file > ).
4. Neoantigen prediction: neoantigens were predicted using the program netMHCpan-3.0^75^. For each patient, we ran netMHCpan-3.0 against their list of neopeptides as many times as the number of their predicted HLA-types (minimum three and maximum six different types per patients) (netMHCpan -p < list_of_peptides_file > -a  < 4-digit_hla_type > > < output_file > ). For each patient we generated 4 (possibly overlapping) lists of neoantigens (Supplementary Data 6): high-affinity binders (<500 nM), strong binders (rank<0.5; note that rank is HLA type-specific), weak binders (rank between 0.5 and 2.0) and strong + weak binders (rank<2.0). Doubles were not counted in any list (i.e. a peptide that was predicted to be a e.g. strong binder for more than one of the patient’s HLA-types was counted only once) and weak binders for an HLA-type were not counted if they also appeared as strong binders for a separate HLA type of the same patient

TCGA ids for the 40 patients for which we predicted neoantigens: TCGA-61-2012, TCGA-61-1995, TCGA-24-2288, TCGA-24-2267, TCGA-24-1474, TCGA-20-0987, TCGA-13-1410, TCGA-09-2051, TCGA-61-2002, TCGA-57-1994, TCGA-29-2427, TCGA-25-2396, TCGA-24-2281, TCGA-24-2261, TCGA-13-1496, TCGA-13-0805, TCGA-13-0795, TCGA-04-1348, TCGA-61-2104, TCGA-61-2094, TCGA-24-2290, TCGA-24-1551, TCGA-23-1123, TCGA-13-0885, TCGA-61-2000, TCGA-59-2351, TCGA-25-1313, TCGA-23-2084, TCGA-23-2079, TCGA-23-2077, TCGA-20-0991, TCGA-13-2060, TCGA-13-0897, TCGA-13-0723, TCGA-09-2044, TCGA-04-1357, TCGA-25-2392, TCGA-13-1484, TCGA-13-0760, TCGA-09-0366.

### Code availability

CRImage is available from Bioconductor. R code for performing relevant analyses are provided as Sweave files for reproducibility online yuanlab.org/software/diversification/sweave.pdf.

## Electronic supplementary material

Supplementary Information

Description of Additional Supplementary Files

Supplementary Data 1

Supplementary Data 2

Supplementary Data 3

Supplementary Data 4

Supplementary Data 5

Supplementary Data 6

## Data Availability

Pathological images and clinicopathological information of the TCGA samples are available in a public repository from the TCGA Data Portal (https://tcga-data.nci.nih.gov/tcga/). All other data supporting the findings of this study are available as part of the reproducible Sweave package.
