# Supplementary material for: Microenvironmental niche divergence shapes BRCA1-dysregulated ovarian cancer morphological plasticity
Source: Nat Commun. 2018 Sep 25;9:3917. doi: 10.1038/s41467-018-06130-3 (PMC6156340; doi:10.1038/s41467-018-06130-3)
Supplement: Supplementary file 1 — Supplementary Information [file 41467_2018_6130_MOESM1_ESM.pdf]

Supplementary Information

Heindl et al.

Microenvironmental niche divergence shapes BRCA1-dysregulated ovarian cancer morphological plasticity

Supplementary Figures

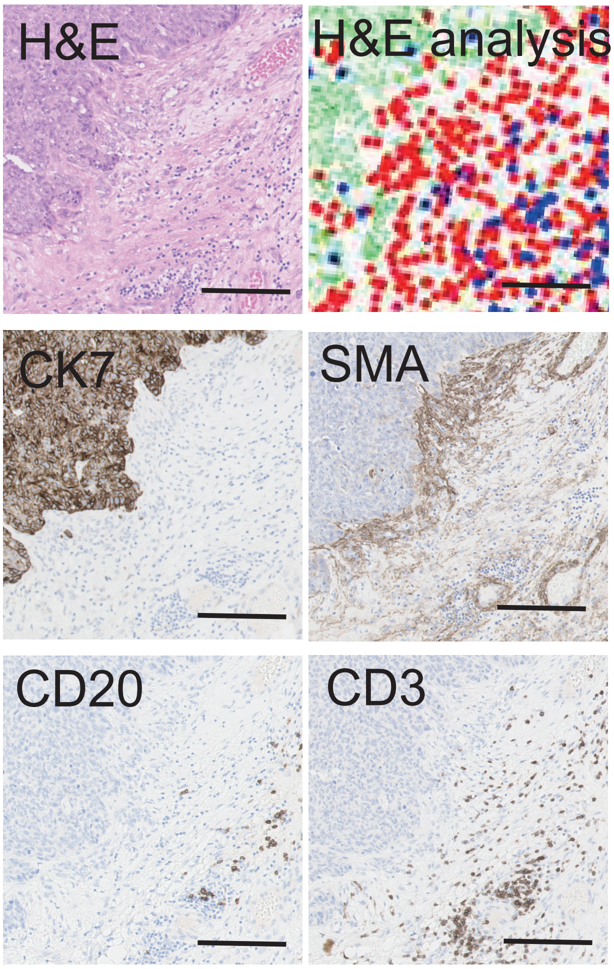

Supplementary Figure 1. Illustrative example of serial sections of immunohistochemistry and H&E.

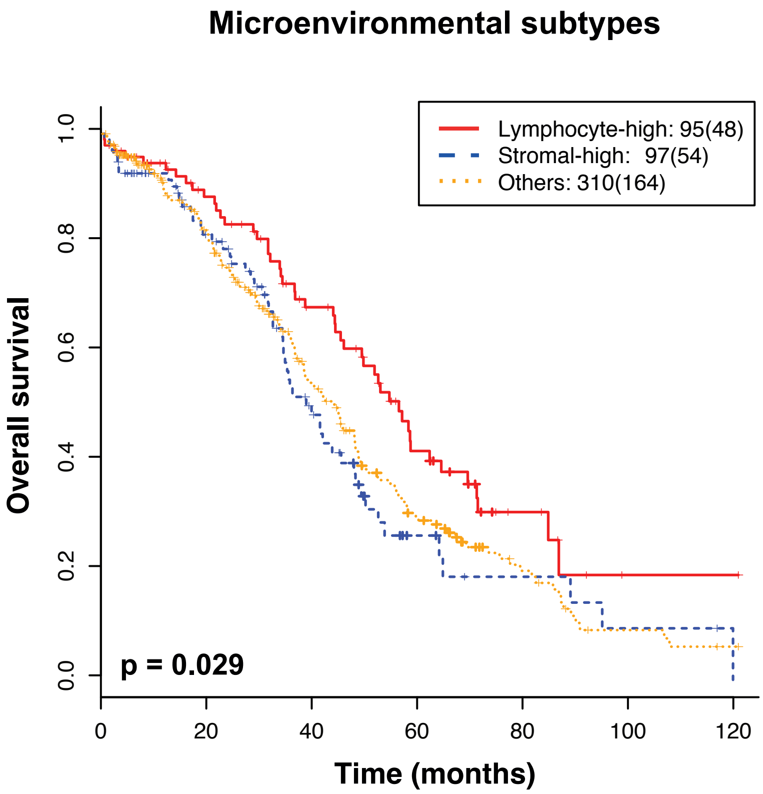

Supplementary Figure 2. Survival analysis of microenvironmental subtypes.

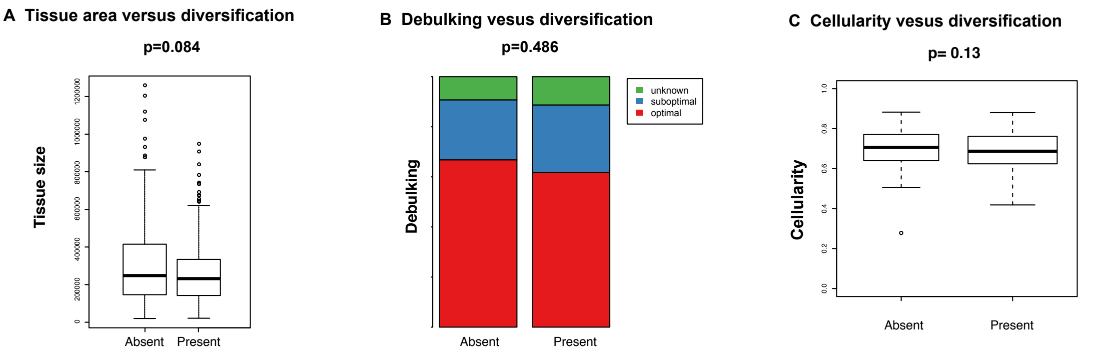

Supplementary Figure 3. Diversification and tissue size and debulking status.

A. Boxplot showing the tissue area (in pixels) for all 514 patients stratified by diversification. B. Barplot illustrates distribution of debulking status stratified by diversification. C. Boxplot illustrating the difference in cellularity stratified by diversification.

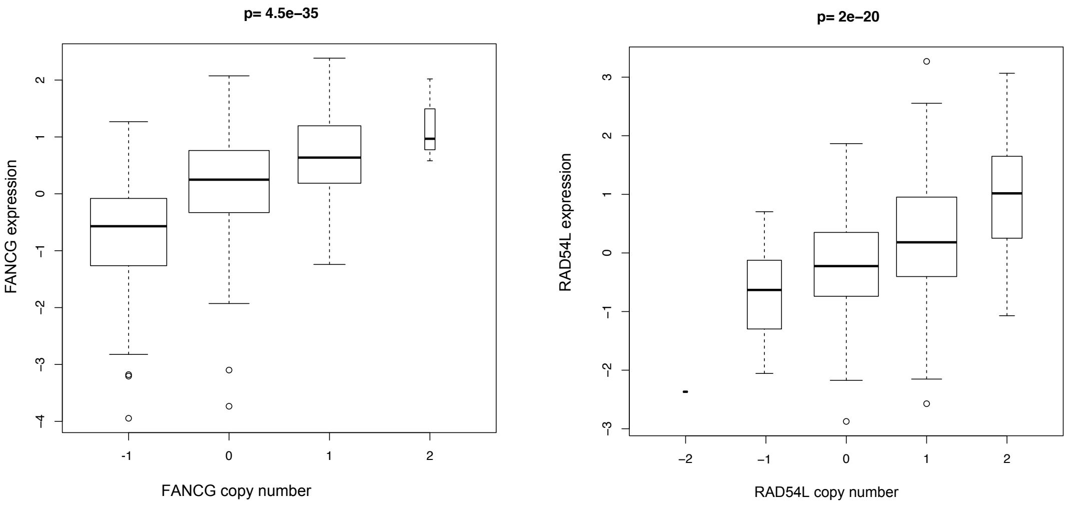

Supplementary Figure 4. Association between copy number and gene expression of FANCG and RAD54L.

BRCA1 mutation and morphological diversification

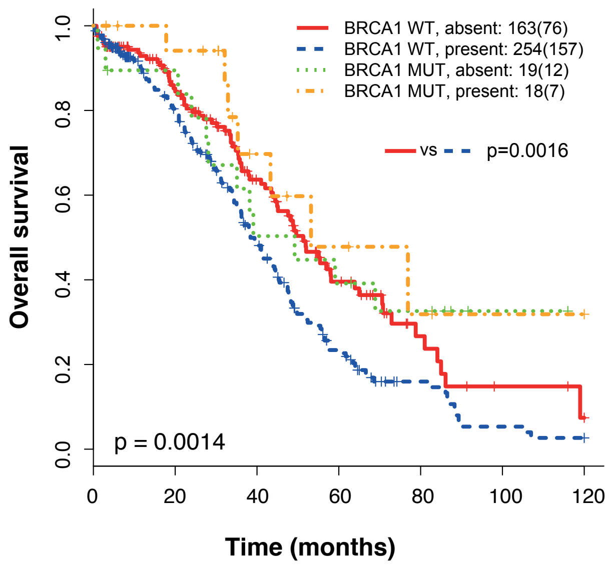

Supplementary Figure 5. Survival analysis of patients stratified by BRCA1 mutation and morphological diversification. Kaplan-Meier plot depicting differences in OS according to *BRCA1* mutation (wildtype WT or mutated MUT) and morphological diversification.

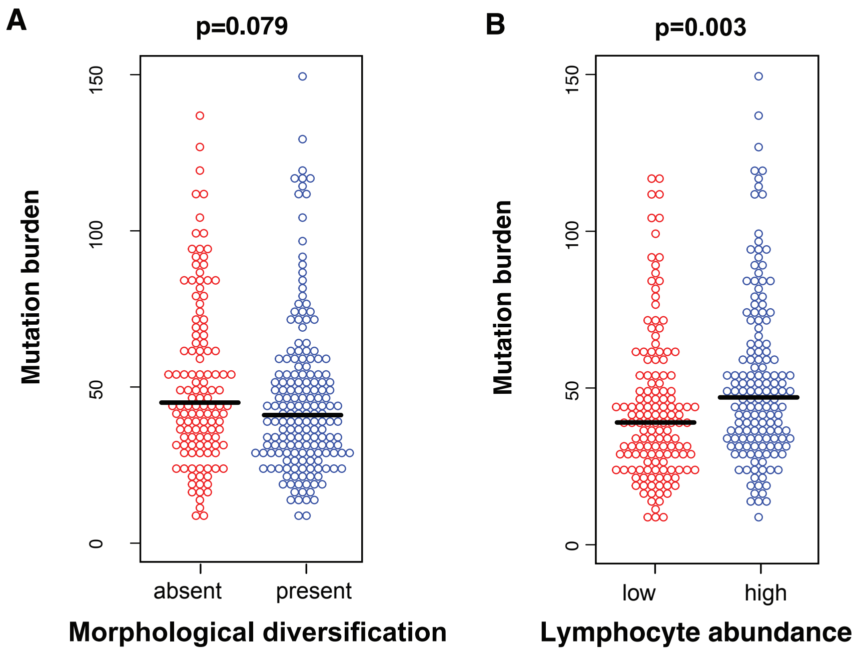

Supplementary Figure 6. Swarmplot to demonstrate the difference in mutation burden according to A. morphological diversification and B. lymphocyte abundance.

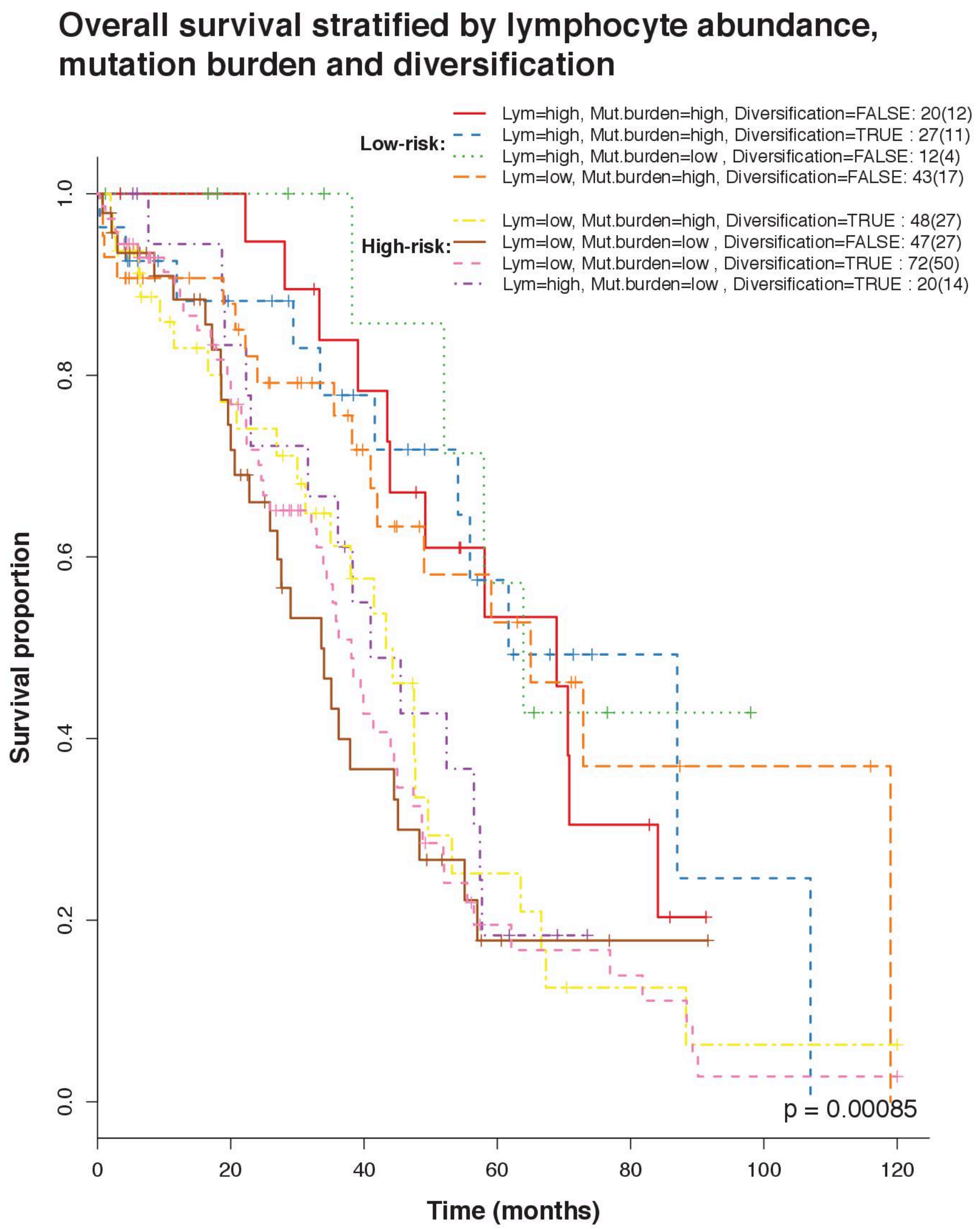

Supplementary Figure 7. Survival analysis of mutation burden, lymphocyte abundance and diversification. Kaplan-Meier curves to demonstrate the differences in OS according to lymphocyte abundance, mutation burden and diversification, revealing two visual groups with different outcomes, which would not have been found without any of the three measures.

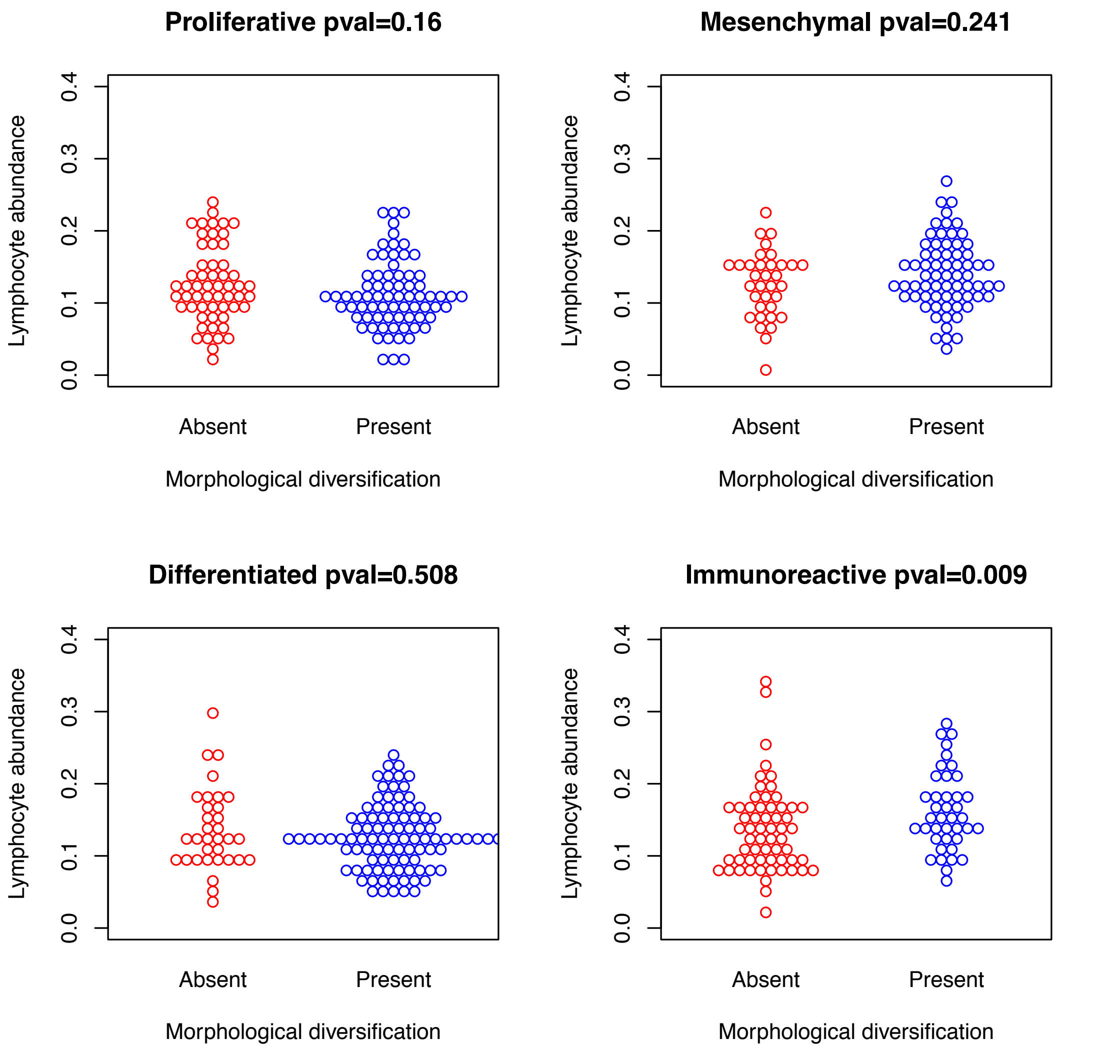

Supplementary Figure 8. Subtype-specific effects on the association between morphological diversification and lymphocyte abundance.

Supplementary Table 1. Enrichment analysis of pathways for genes correlated with abundance of cancer cells, lymphocytes and stromal cells.

Cell Type	Universe Size	Expected Hits	Observed Hits	Gene Set Size	Adjusted Pvalue	Gene Set Term
	1	0.763118912	9	34	5.40E-05	KEGG_DNA_REPLICATION
	2	0.650895542	8	29	0.00011	REACTOME_DNA_STRAND_ELONGATION
	3	0.740674238	8	33	0.00022	REACTOME_ACTIVATION_OF_ATR_IN_RESPONSE_TO_REPLICATION_STRESS
	4	0.875342281	8	39	0.00064	REACTOME_G2_M_CHECKPOINTS
**Cancer**	5	0.650895542	7	29	7.00E-04	REACTOME_ACTIVATION_OF_THE_PRE_REPLICATIVE_COMPLEX
	6	1.279346411	9	57	0.00097	REACTOME_TELOMERE_MAINTENANCE
	7	0.314225434	5	14	0.0015	REACTOME_REPAIR_SYNTHESIS_FOR_GAP_FILLING_BY_DNA_POL_IN_TC_NER
	8	*2.109799345*	*11*	*94*	*0.0015*	*REACTOME_CHROMOSOME_MAINTENANCE*
	9	*7.743412488*	*22*	*345*	*0.0018*	*REACTOME_CELL_CYCLE*
	10	0.583561521	6	26	0.0022	REACTOME_EXTENSION_OF_TELOMERES
	1	3.725815864	15	166	0.0012	KEGG_CHEMOKINE_SIGNALING_PATHWAY
	2	4.219598689	16	188	0.0012	KEGG_FOCAL_ADHESION
	3	2.379135431	12	106	0.0012	KEGG_LEUKOCYTE_TRANSENDOTHELIAL_MIGRATION
	4	0.673340216	7	30	0.0012	PID_INTEGRIN_A4B1_PATHWAY
	5	2.289356736	12	102	0.0012	REACTOME_SIGNALING_BY_ILS
**Immune**	6	0.965120977	8	43	0.0012	REACTOME_IL_3_5_AND_GM_CSF_SIGNALING
	7	2.087354671	11	93	0.0014	PID_CXCR4_PATHWAY
	8	1.054899672	8	47	0.0014	PID_PI3KCI_PATHWAY
	9	5.139830318	17	229	0.0024	KEGG_CYTOKINE_CYTOKINE_RECEPTOR_INTERACTION
	10	11.62634107	28	518	0.0024	REACTOME_SIGNALING_BY_GPCR
	1	16.99061813	62	757	1.20E-15	NABA_MATRISOME
	2	4.488934776	27	200	5.10E-11	NABA_CORE_MATRISOME
	3	1.683350541	15	75	4.20E-08	REACTOME_EXTRACELLULAR_MATRIX_ORGANIZATION
	4	1.010010325	12	45	6.60E-08	PID_SYNDECAN_1_PATHWAY
**Stromal**	5	5.117385644	22	228	2.60E-06	REACTOME_AXON_GUIDANCE
	6	3.074920321	17	137	2.60E-06	NABA_ECM_GLYCOPROTEINS
	7	7.878080531	27	351	5.00E-06	REACTOME_DEVELOPMENTAL_BIOLOGY
	8	0.740674238	9	33	5.00E-06	NABA_COLLAGENS
	9	12.50168335	35	557	7.10E-06	NABA_MATRISOME_ASSOCIATED
	10	1.077344346	10	48	1.10E-05	REACTOME_COLLAGEN_FORMATION
