# Supplementary material for: Microenvironmental niche divergence shapes BRCA1-dysregulated ovarian cancer morphological plasticity
Source: Nat Commun. 2018 Sep 25;9:3917. doi: 10.1038/s41467-018-06130-3 (PMC6156340; doi:10.1038/s41467-018-06130-3)
Supplement: Supplementary file 2 — Description of Additional Supplementary Files [file 41467_2018_6130_MOESM2_ESM.pdf]

Supplementary Data:

Supplementary Data 1. Immunohistochemistry scores for H&E image analysis validation. Each row is a sample and columns denote the following information: Cancer, Lym, Stromal: percentage of cells from H&E calculated by averaging regional scores; CK7, CD3, SMA: sample-level scores from IHC calculated as the average of regional scores; CK7cancer, CD3lym and SMAstromal: spatial correlation between IHC CK7/CD3/SMA and H&E-based estimate of cancer/lymphocyte/stromal abundance; CD3Gal3: spatial correlation between CD3 and galectin-3 expression; RegVal_HECK7/CD3/SMA/Average/std: DICE coefficient measuring the overlap between HE and registered CD7, CD3 and SMA, their average and standard deviation. DIV_gal3: average galectin-3 expression in diversified regions; DIVnot_gal3: average galectin-3 expression in non-diversifying regions. NaN: no diversification region detected.

Supplementary Data 2. Differential gene expression analysis results.

Supplementary Data 3. Enrichment analysis of genes differentially expressed in the diversified samples.

Supplementary Data 4. Copy number enrichment analysis result.

Supplementary Data 5. Methylation data analysis result.

Supplementary Data 6. A summary of calculated somatic mutational and neoantigen load for the 40 patients with data available in the Immunoreactive subtype. (ID=TCGA patient ID; DIVER=whether tumor morphological diversification is observed (TRUE) or not (FALSE); #MUTS_TCGA=(somatic)mutational load as calculated by TCGA; #MUTS=(somatic)mutational load as calculated by us with the variant calling protocol described above (includes: missense, inframe_insertion, inframe_deletion, frameshift, stop_lost and stop_gained variants); #STRONG_B=neoantigen load according to predicted strong binders (rank-based); #WEAK_B= neoantigen load according to predicted weak binders (rank-based); #STRONG_B+#WEAK_B=neoantigen load according to predicted strong binders plus predicted weak binders (rank-based); #
